# Burnout, depression, and suicidal ideation among Chinese hospital physicians: prevalence and the distinct roles of research and teaching pressure

**DOI:** 10.7189/jogh.16.04323

**Published:** 2026-10-23

**Authors:** Xingyu Bao, Yanan Zhao, Tingkun Yang, Ping Huang, Dan Guo

**Affiliations:** 1 Faculty of Health and Wellness, City University of Macau, Macau SAR, China; 2 Department of Child Health Care, Luzhou People’s Hospital, Luzhou, China; 3 Department of Scientific Research and Teaching, Beijing Genertec Aerospace Hospital, Beijing, China

**Keywords:** burnout, depression, suicidal ideation, physicians, epidemiology, cross-sectional study

## Abstract

**Background:**

Physician mental health is a global concern, but comprehensive data from China, the home to one of the world's largest medical workforces, remains limited. We assessed the prevalence of burnout, depression, and suicidal ideation among Chinese hospital physicians and examined their comorbidity patterns, instrument concordance, and associated occupational factors.

**Methods:**

We conducted a cross-sectional study from September to November 2025 across four selected regions in China using purposive sampling. A total of 961 physicians completed validated mental health assessments. Burnout was assessed with the Professional Fulfillment Index (PFI) as the primary instrument and the Maslach Burnout Inventory–Human Services Survey (MBI-HSS) for cross-validation. We measured depression using the four-item Patient-Reported Outcomes Measurement Information System (PROMIS) Depression Short Form (T-scores calibrated to the USA reference population), and past-year suicidal ideation with a validated single-item measure. We used multivariable logistic regression to identify independent correlates of each outcome.

**Results:**

Of 961 physicians, 644 (67.0%) met PFI criteria for high burnout and 529 (55.0%) met MBI-HSS criteria; instrument agreement was moderate (Cohen's kappa = 0.55). Moderate-to-severe depression (PROMIS T-score ≥60) affected 363 (37.8%) physicians, and 41 (4.3%) reported past-year suicidal ideation. Research pressure and teaching pressure were both independently associated with burnout but showed divergent patterns: research pressure was associated with emotional exhaustion and appeared more evident among junior physicians, whereas teaching pressure was associated with depersonalisation and was significantly associated with burnout among senior physicians in Fujian-inclusive analyses. Income satisfaction was the strongest modifiable protective factor against both burnout and depression.

**Conclusions:**

In this purposive multi-region sample, burnout and depression were common, and prevalence estimates were instrument dependent. The divergent association patterns of research and teaching pressure across burnout dimensions and career stages suggest that future studies should test whether targeted, stage-specific institutional reforms are warranted. Income dissatisfaction warrants attention as a potentially modifiable risk factor in future longitudinal research.

Physician burnout has reached epidemic proportions globally, with previous studies and a systematic review indicating that approximately 50% of practising physicians experience burnout symptoms [1–3]. Characterised by emotional exhaustion, depersonalisation, and reduced sense of professional accomplishment, burnout is now recognised by the World Health Organization as an occupational syndrome with serious consequences for both individual physicians and healthcare delivery [4]. Furthermore, the elevated rates of depression and suicidal ideation among physicians pose direct threats to both occupational well-being and clinical safety [5,6].

China’s physician workforce is among the largest and most strained in the world. Yet China remains systematically underrepresented in the global burnout literature, which has been dominated by studies from North America and Western Europe. Evidence from high-income healthcare systems may not translate directly to China, where unique structural pressures further intensify physician mental health risk. Chinese doctors in major tertiary hospitals frequently manage patient volumes that substantially exceed international norms [7], with outpatient consultations commonly lasting <10 minutes per patient. Chinese academic physicians face a triple burden: high patient volumes, research productivity requirements, and formal teaching responsibilities for medical students, residents, and graduate trainees – all without adequate protected time or compensation. The academic promotion system further intensifies this pressure by demanding research publication output on top of already overwhelming clinical demands [8,9]. Physicians also face relatively low salaries despite long working hours [10].

Despite these concerning conditions, comprehensive epidemiological data on Chinese physician mental health remain limited. Most prior studies have focused on single institutions or specific specialities, used heterogeneous burnout definitions that preclude cross-study comparability, and rarely assessed burnout, depression, and suicidal ideation simultaneously in geographically diverse samples [11,12]. Moreover, no study has systematically compared burnout assessment instruments to evaluate measurement concordance in China– a critical methodological gap given that scores from different burnout measures cannot currently be directly compared across studies [13], and that ongoing debates about the conceptual boundaries of burnout and its overlap with related constructs such as depression and anxiety further underscore the importance of measurement validity in this field [14,15]. Furthermore, while research and teaching pressure have been identified as potential stressors in academic medicine, it remains unknown whether their effects differ by gender or career stage – a critical gap for designing targeted interventions.

Rather than estimating nationally representative prevalence, we aimed to examine the correlates and mechanisms of physician burnout across diverse regional and institutional contexts in China. We addressed three questions. First, whether the Professional Fulfilment Index (PFI) and Maslach Burnout Inventory–Human Services Survey (MBI-HSS) yield comparable burnout classifications and risk factor associations in a non-Western clinical population – a prerequisite for meaningful cross-national comparison. Second, what is the observed burden of burnout, depression, and suicidal ideation among physicians in this purposive multi-region sample, and to what extent do these conditions co-occur? Third, are research pressure and teaching pressure associated with different burnout dimensions, potentially suggesting distinct role-related pathways? We examined whether the PFI and MBI-HSS yielded comparable burnout classifications in this Chinese physician sample, given their differences in construct definition, response format, and cut-off derivation. We also hypothesised that burnout, depression, and suicidal ideation would be common in this sample, and that research and teaching pressure would emerge as independent burnout predictors with differential effects by career stage. As an exploratory question, we additionally examined whether generative artificial intelligence (AI) tool use was associated with physician mental health outcomes.

## METHODS

### Study design and participants

We conducted this cross-sectional study from September to November 2025 in four selected regions of China. We used a purposive maximum-variation sampling strategy rather than probability sampling. We selected regions based on geographic location, economic development level, and feasibility of institutional access through existing research collaborations. We aimed to include contrasting regional and hospital practice contexts, not to generate a nationally representative sample of Chinese physicians. The selected regions were: Fengtai District, Beijing (gross domestic product (GDP) per capita: CNY 200,278 in 2023; 2.2 times the national average), representing a highly developed metropolitan area with concentrated medical resources; Taiyuan, Shanxi Province (GDP per capita: CNY 102,922; 1.1 times the national average), representing a central provincial capital with a transitioning industrial economy; Luzhou, Sichuan Province (GDP per capita: CNY 63,913 in 2023; 0.72 times the national average), representing a southwestern developing regional healthcare context; and Ningde, Fujian Province (GDP per capita: CNY 120,619; 1.3 times the national average), representing a southeastern coastal city experiencing rapid economic growth. We obtained economic development data from the National Bureau of Statistics of China. Consistent with purposive sampling methodology, we recruited participants through existing institutional partnerships and snowball referrals within each region, with local research coordinators facilitating survey distribution. Accordingly, the achieved sample should be interpreted as a multi-region institutional sample shaped by access to collaborating hospitals, rather than as a probability-based sample of all Chinese hospital physicians. Because institutional access and participation rates varied across sites, the achieved sample was not proportionally distributed across regions, with Fujian contributing the largest subgroup (n = 619; 64.4%) (Table S1 in the **Online Supplementary Document**). To assess whether the Fujian subsample influenced the findings, we conducted a geographic sensitivity analysis excluding all Fujian participants (n = 342).

Participating hospitals included tertiary and secondary general hospitals. Eligible participants were physicians actively practising clinical medicine at these institutions. We excluded physicians on extended leave (>3 months), retired, or exclusively engaged in administrative roles. We approached 1,142 physicians who initiated survey participation; of these, we excluded 181 for incomplete responses or ineligible department or regional affiliation, yielding a final analytical sample of 961 physicians (Figure S1 in the **Online Supplementary Document**). Because survey access required active opt-in through an institutional platform, we did not capture non-initiators in the recruitment denominator, so we could not calculate a conventional response rate. We calculated the sample size *a priori* based on an estimated burnout prevalence of 50% among physicians [1], a 5% margin of error, and a 95% confidence interval (CI), yielding a minimum required sample of 384 participants. The achieved sample of 961 physicians substantially exceeded this requirement and provided sufficient outcome events for the primary multivariable analyses of common outcomes, including PFI-defined burnout and moderate-to-severe depression. Because suicidal ideation was reported by only 41 participants and subgroup analyses involved smaller strata, these analyses were considered exploratory and were interpreted with caution. Ultimately, 619 (64.4%) participants were from Fujian, 127 (13.2%) from Sichuan, 118 (12.3%) from Beijing, and 97 (10.1%) from Shanxi.

### Measures

We assessed burnout using two validated instruments to enable comparison and evaluate measurement concordance. The PFI includes a ten-item burnout subscale comprising work exhaustion (four items) and interpersonal disengagement (six items), with items scored on a five-point Likert scale from ‘not at all’ (0) to ‘extremely’ (4) [16]. We defined high burnout as a mean score ≥1.33, the established cut-off validated in previous physician samples [16,17]. We also administered the MBI-HSS, validated for use in Chinese healthcare workers, to cross-validate PFI-based classifications. The MBI-HSS assesses three dimensions – emotional exhaustion, depersonalisation, and personal accomplishment – using a seven-point frequency scale from ‘never’ (0) to ‘every day’ (6). High burnout was defined as meeting at least one published dimension threshold (emotional exhaustion ≥27 or depersonalisation ≥10) [1].

Depression was assessed using the Patient-Reported Outcomes Measurement Information System (PROMIS) Depression Short Form (four-item), which assesses core depressive symptoms (*i.e.* feeling worthless, helpless, depressed, hopeless) using a five-point frequency scale from ‘never’ (1) to ‘always’ (5) [18–20]. We converted raw scores to T-scores using PROMIS scoring tables, with moderate-to-severe depression defined as T≥60, consistent with published guidelines for clinically significant symptoms [19,20].

We assessed suicidal ideation with the question: ‘During the past 12 months, have you had thoughts of taking your own life?’ (yes/no), which has demonstrated concurrent validity with comprehensive assessments and has been used extensively in previous physician studies [18,21,22].

We collected demographic variables including age (≤35, 36–45, ≥46 years), sex (male, female), and marital and parental status (married with children, married without children, divorced/separated/single). Professional characteristics included professional title (resident, attending physician, associate chief physician, chief physician, other), years of practice (<5, 5–10, 11–15, 16–20, >20 years), and department/specialty. Department classification was based on established burnout research and the Job Demands-Resources model [23–26], grouping specialties by similar patterns of job demands and work characteristics into type 1 (*i.e.* high-acuity emergency settings: emergency medicine, intensive care unit/critical care), type 2 (*i.e.* surgical/procedural specialties: general surgery, orthopaedics, neurosurgery, urology, cardiothoracic surgery), type 3 (*i.e.* complex chronic care: internal medicine subspecialties, oncology), type 4 (*i.e.* mother-child health: obstetrics/gynaecology, paediatrics, neonatology), type 5 (*i.e.* diagnostic/supportive services: radiology, pathology, laboratory medicine, anaesthesiology), and type 6 (*i.e.* other specialties: dermatology, ophthalmology, otolaryngology, dentistry, rehabilitation, psychiatry, traditional Chinese medicine). This classification aligns with national burnout epidemiology data from China showing that acute care, internal medicine, oncology, and psychiatry carry significantly elevated burnout risk relative to other specialities [7,27]. We used these department categories to reduce dimensionality and reflect broad work-demand profiles. They were not intended to imply empirical homogeneity within each category.

We also collected work environment factors including night shifts (yes/no), weekly patient volume (<10, 10–30, 30–50, >50 patients), and geographic region (Beijing, Fujian, Sichuan, Shanxi). Organisational factors included income satisfaction (five-point scale from ‘very dissatisfied’ to ‘very satisfied’), perceived research pressure, and perceived teaching pressure. We assessed research pressure and teaching pressure using single binary self-report items indicating whether participants perceived high pressure in each domain. These items were intended to capture domain-specific perceived academic job demands rather than detailed objective workload indicators such as publication requirements, grant expectations, teaching hours, supervisory load, or protected time. This approach is consistent with large-scale physician survey practice [1,28], and supported by evidence that single-item measures can adequately capture conceptually discrete perceived job demands [29].

As an exploratory variable, we assessed generative AI tool use with a single item dichotomised into user *vs.* non-user categories.

### Statistical analysis

Multivariable logistic regression models were specified for three separate outcomes: high burnout (PFI ≥ 1.33), moderate-to-severe depression (PROMIS T-score ≥60), and suicidal ideation (yes *vs.* no). Covariates were entered in blocks: demographic factors (*i.e.* age, sex, marital status), professional factors (*i.e.* training status, professional title, department type, region), work environment factors (*i.e.* night shifts, patient volume), and organisational factors (*i.e.* research pressure, teaching pressure, income satisfaction). For suicidal ideation, we additionally fitted a reduced logistic regression model including age group, sex, income satisfaction, research pressure, and teaching pressure to reduce overfitting risk.

To examine whether associations between research and teaching pressure and burnout varied by key demographic and professional characteristics, we conducted pre-specified subgroup analyses stratified by sex and professional title. Sex was selected based on well-documented gender differences in physician burnout. We chose professional title as a proxy for career stage, reflecting distinct role expectations – junior physicians face intense pressure to publish for promotion, while senior physicians bear primary responsibility for clinical supervision and trainee education. In each stratum, we fitted fully adjusted logistic regression models identical to the primary analysis, with research pressure and teaching pressure as the primary exposures and PFI-defined burnout as the outcome. To assess formal evidence of effect modification, we additionally tested interaction terms between research pressure or teaching pressure and sex or professional title group in separate multivariable logistic regression models adjusted for the same covariates as the primary burnout model.

We also conducted several sensitivity analyses, including linear regression treating PFI burnout score and PROMIS T-score as continuous outcomes, geographic restriction excluding Fujian province, and exclusion of physicians with the ‘other’ professional title category (n = 814) to assess whether results were driven by the heterogeneous ‘other’ group. All sensitivity analyses additionally re-ran primary models using the MBI-HSS as an alternative outcome to assess cross-instrument consistency.

We used SPSS, version 26.0 (IBM Corp., Armonk, New York, USA) for all analyses, with two-tailed *P*-values <0.05 considered statistically significant.

## RESULTS

### Participant characteristics

Of the 961 physicians, 497 (51.7%) were female, and 464 (48.3%) were male (**Table 1**). The majority were aged ≤35 years (51.5%), married with children (60.7%), and practised in Fujian province (64.4%).

**Table 1:** Characteristics of Chinese physicians stratified by PFI burnout status*

	Total (n = 961)	High burnout (n = 644)	Low burnout (n = 317)	*P*-value†
**Age group in years**				0.645
≤35	495 (51.5)	325 (50.5)	170 (53.6)	
36–45	310 (32.3)	213 (33.1)	97 (30.6)	
≥46	156 (16.2)	106 (16.5)	50 (15.8)	
**Sex**				<0.001
Male	464 (48.3)	339 (52.6)	125 (39.4)	
Female	497 (51.7)	305 (47.4)	192 (60.6)	
**Marital and parental status**				0.022
Married with children	583 (60.7)	402 (62.4)	181 (57.1)	
Married without children	63 (6.6)	48 (7.5)	15 (4.7)	
Divorced/single	315 (32.8)	194 (30.1)	121 (38.2)	
**Professional title**				0.009
Resident	278 (28.9)	179 (27.8)	99 (31.2)	
Attending physician	253 (26.3)	189 (29.3)	64 (20.2)	
Associate chief physician	196 (20.4)	134 (20.8)	62 (19.6)	
Chief physician	87 (9.1)	57 (8.9)	30 (9.5)	
Others	147 (15.3)	85 (13.2)	62 (19.6)	
**Years of practice**				0.173
<5	297 (30.9)	189 (29.3)	108 (34.1)	
5–10	200 (20.8)	138 (21.4)	62 (19.6)	
11–15	177 (18.4)	130 (20.2)	47 (14.8)	
16–20	119 (12.4)	73 (11.3)	46 (14.5)	
>20	168 (17.5)	114 (17.7)	64 (17.0)	
**Department type**				0.228
High acuity emergency settings	59 (6.1)	41 (6.4)	18 (5.7)	
Surgical procedural specialities	173 (18.0)	115 (17.9)	58 (18.3)	
Complex chronic care	218 (22.7)	162 (25.2)	56 (17.7)	
Mother-child health	171 (17.8)	105 (16.3)	66 (20.8)	
Diagnostic supportive services	101 (10.5)	73 (11.3)	28 (8.8)	
Other	239 (24.9)	148 (23.0)	91 (28.7)	
**Region**				<0.001
Fujian	619 (64.4)	441 (68.5)	178 (56.2)	
Beijing	118 (12.3)	60 (9.3)	58 (18.3)	
Sichuan	127 (13.2)	79 (12.3)	48 (15.1)	
Shanxi	97 (10.1)	64 (9.9)	33 (10.4)	
**Night shift rotation**				<0.001
Required to work night shifts	766 (79.7)	536 (83.2)	230 (72.6)	
No night shifts	195 (20.3)	108 (16.8)	87 (27.4)	
**Research pressure**				<0.001
High pressure	218 (22.7)	177 (27.5)	41 (12.9)	
No/low pressure	743 (77.3)	467 (72.5)	276 (87.1)	
**Teaching pressure**				<0.001
High pressure	80 (8.3)	71 (11.0)	9 (2.8)	
No/low pressure	881 (91.7)	573 (89.0)	308 (97.2)	
**Weekly patient volume**				0.102
<10	188 (19.6)	119 (18.5)	69 (21.8)	
10–30	258 (26.8)	185 (28.7)	73 (23.0)	
30–50	157 (16.3)	114 (17.7)	43 (13.6)	
>50	358 (37.3)	226 (35.1)	132 (41.6)	
**Income satisfaction**				<0.001
Dissatisfied	233 (24.2)	187 (29.0)	46 (14.5)	
Neutral	385 (40.1)	278 (43.2)	107 (33.8)	
Satisfied	343 (35.7)	179 (27.8)	164 (51.7)	
**AI tool use**				0.019
Yes	586 (61.0)	376 (58.4)	210 (66.2)	
No	375 (39.0)	268 (41.6)	107 (33.8)	

AI – artificial intelligence, PFI – Professional Fulfilment Index

*Values are presented as n (%) and percentages may not total 100 because of rounding.

†*P*-values are from a χ^2^ test for categorical variables.

Compared with physicians with low burnout, those with high burnout were more likely to be male (52.6% *vs.* 39.4%; *P* < 0.001), married with children (62.4% *vs.* 57.1%; *P* = 0.022), and hold attending physician titles (29.3% *vs.* 20.2%; *P* = 0.009). High burnout physicians also reported higher rates of night shift work (83.2% *vs.* 72.6%; *P* < 0.001), high research pressure (27.5% *vs.* 12.9%; *P* < 0.001), high teaching pressure (11.0% *vs.* 2.8%; *P* < 0.001), and income dissatisfaction (29.0% *vs.* 14.5%; *P* < 0.001). Conversely, AI tool use was lower among physicians with high burnout than those with low burnout (58.4% *vs.* 66.2%; *P* = 0.019). We found no significant differences by age group, years of practice, department type, or weekly patient volume.

Burnout rates did not differ significantly by age group (*P* = 0.645). By department type, burnout prevalence varied across department types, with the highest rates observed in the internal medicine and imaging/radiology departments (Table S2 in the **Online Supplementary Document**).

### Prevalence of mental health conditions

Using the PFI, 644 (67.0%) physicians met criteria for high burnout (**Table 2**). In contrast, the MBI-HSS identified 529 (55.0%) physicians as experiencing high burnout based on meeting at least one dimension threshold. Agreement between the two instruments was moderate (Cohen's kappa = 0.55; 95% CI = 0.50–0.60), with 482 (50.2%) physicians classified as burned out by both measures and 162 (16.9%) by PFI only. Internal consistency was excellent for both the PFI burnout subscale (Cronbach α = 0.93) and the MBI-HSS subscales (Cronbach α = 0.88–0.93). Moderate-to-severe depression (PROMIS T-score ≥60) affected 363 (37.8%; 95% CI = 34.7–40.9) physicians. Past 12-month suicidal ideation was reported by 41 (4.3%; 95% CI = 3.1–5.5) physicians.

**Table 2:** Prevalence of mental health conditions and comorbidity patterns among Chinese physicians (n = 961)

	n	% (95% CI)
**PFI burnout**		
High burnout (≥1.33)	644	67.0 (64.0–70.0)
**MBI-HSS burnout**		
High emotional exhaustion (≥27)	451	46.9 (43.7–50.1)
High depersonalisation (≥10)	409	42.6 (39.5–45.7)
High burnout (≥1 dimension)	529	55.0 (51.9–58.2)
**Concordance between PFI and MBI**		
Both instruments high	482	50.2 (47.0–53.4)
PFI high only	162	16.9 (14.5–19.3)
MBI high only	47	4.9 (3.5–6.3)
Both instruments low	270	28.1 (25.3–30.9)
**Depression**		
Normal (*t* < 55)	320	33.3 (30.3–36.3)
Mild (*t* = 55–59.9)	278	28.9 (26.0–31.8)
Moderate (*t* = 60–69.9)	336	35.0 (32.0–38.0)
Severe (T≥70)	27	2.8 (1.8–3.8)
Moderate to severe (T≥60)	363	37.8 (34.7–40.9)
**Suicidal ideation**		
Yes	41	4.3 (3.1–5.5)
Comorbidity patterns*		
None of the conditions	298	31.0 (28.1–34.0)
**Single condition**		
Burnout only	292	30.4 (27.5–33.3)
Depression only	16	1.7 (0.9–2.5)
Suicidal ideation only	3	0.3 (0.0–0.7)
**Two conditions**		
Burnout + depression	314	32.7 (29.7–35.7)
Burnout + suicidal ideation	5	0.5 (0.1–1.0)
Depression + suicidal ideation	0	0 (0.0–0.4)
**All 3 conditions**	33	3.4 (2.2–4.7)

CI – confidence interval, MBI-HSS – Maslach Burnout Inventory–Human Services Survey, PFI – Professional Fulfilment Index, PROMIS – Patient-Reported Outcomes Measurement Information System

*Based on high burnout (PFI≥1.33), moderate-to-severe depression (PROMIS T≥60), and suicidal ideation (yes).

Comorbidity patterns revealed substantial overlap between conditions. Burnout and depression co-occurred in 314 (32.7%) physicians, while all three conditions – burnout, depression, and suicidal ideation – co-occurred in 33 (3.4%) physicians. Among the 41 physicians reporting suicidal ideation, 38 (92.7%) also had burnout, and 33 (80.5%) also had depression, indicating that suicidal ideation rarely occurred in isolation.

### Multivariable logistic regression analyses

In the multivariable model for high burnout, female sex was associated with 28% lower odds of burnout (odds ratio (OR) = 0.72; 95% CI = 0.52–0.99) compared to male sex (**Figure 1**; Table S3 in the **Online Supplementary Document**). Required night shift work was associated with increased burnout risk (OR = 1.61; 95% CI = 1.08–2.41). Compared to those dissatisfied with their income, satisfied physicians had 76% lower odds of burnout (OR = 0.24; 95% CI = 0.16–0.38). Both teaching pressure (OR = 2.29; 95% CI = 1.40–5.03) and research pressure (OR = 2.17; 95% CI = 1.42–3.31) were independently associated with approximately doubled odds of burnout. Geographic variation was observed, with physicians in Beijing (OR = 0.39; 95% CI = 0.24–0.63) showing lower burnout odds than those in Fujian. The use of AI tools was not significantly associated with burnout.

**Figure 1:**
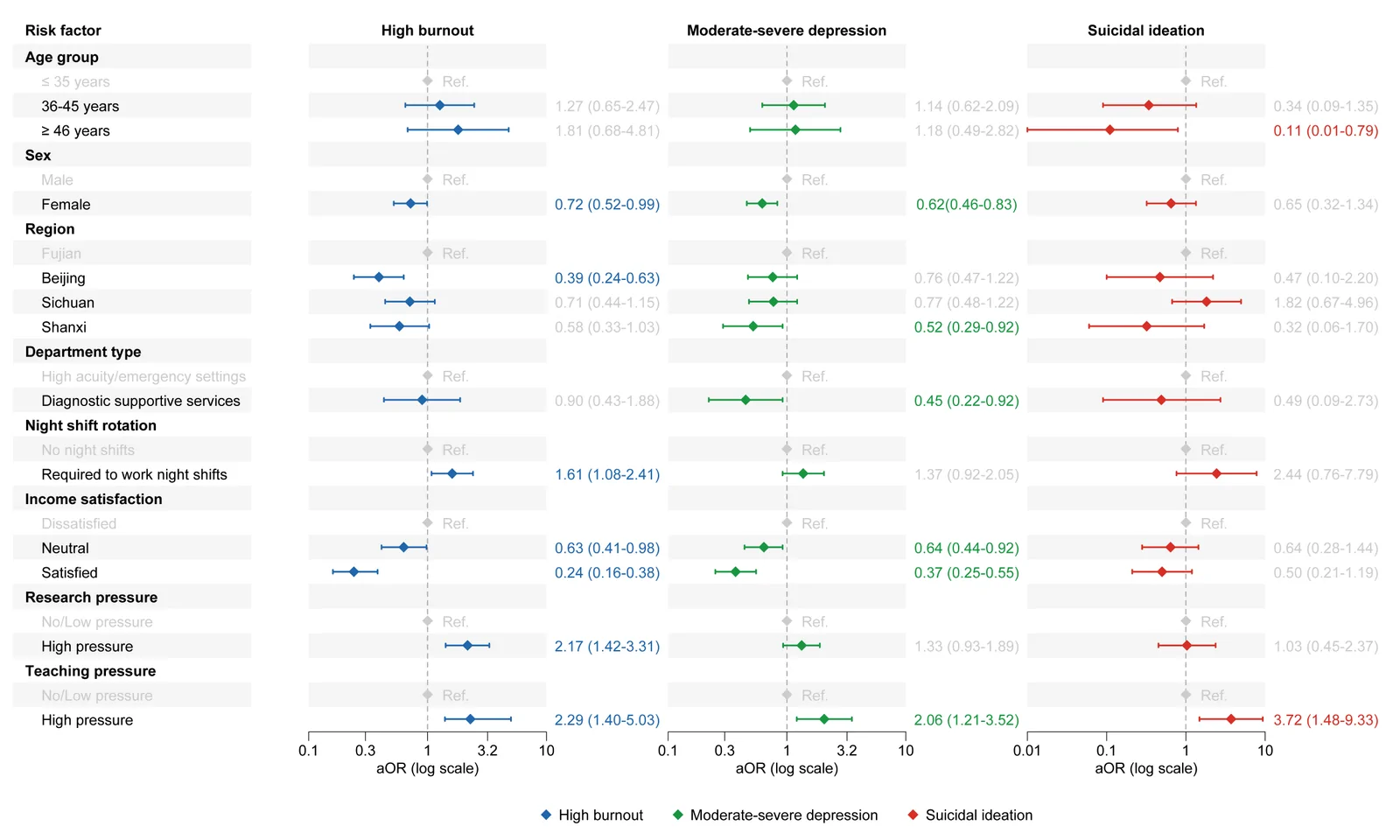
Adjusted associations between significant risk factors and mental health outcomes among Chinese physicians (n = 961). All models adjusted for age, sex, marital/parental status, region, professional title, years of practice, department type, weekly patient volume, night shift rotation, income satisfaction, research pressure, teaching pressure, and AI tool use. Only risk factors with at least one statistically significant association (*P* < 0.05) across the three outcomes are displayed. aOR – adjusted odds ratio.

In the multivariable model for moderate-to-severe depression, female sex was associated with lower odds of depression (OR = 0.62; 95% CI = 0.46–0.83). Compared to those dissatisfied with their income, satisfied physicians had lower odds of depression (OR = 0.37; 95% CI = 0.25–0.55). Teaching pressure was significantly associated with depression (OR = 2.06; 95% CI = 1.21–3.52), while research pressure showed a positive but non-significant trend (OR = 1.33; 95% CI = 0.93–1.89). Night shift work and AI tool use were not significantly associated with depression.

We also analysed risk factors of suicidal ideation (n = 41). To reduce overfitting risk, we additionally fitted a reduced model including age group, sex, income satisfaction, research pressure, and teaching pressure (Table S4 in the **Online Supplementary Document**). In this reduced model, teaching pressure remained positively associated with suicidal ideation (OR = 3.61; 95% CI = 1.50–8.71), whereas age group, sex, income satisfaction, and research pressure were not statistically significant. Because of the limited sample size and potential sparse-data instability, this analysis should be considered exploratory, and the findings should be interpreted cautiously.

### Comparison of risk factor associations across burnout instruments

Despite differences in prevalence estimates, the pattern of risk factor associations was highly consistent across PFI burnout and the three MBI-HSS dimensions (**Table 3**). Female sex was protective across all outcomes (OR range = 0.53–0.72). Night shift work (OR range = 1.61–2.09), income satisfaction (OR range = 0.20–0.42), and research pressure (OR range = 1.42–2.17) showed directionally consistent associations across all measures.

**Table 3:** Comparison of associations between risk factors and burnout across measurement instruments

		MBI-HSS, OR (95% CI)		
	**PFI, OR (95% CI)**	**Exhaustion**	**Depersonalisation**	**Composite**
**Age group in years**				
≤35	ref	ref	ref	ref
36–45	1.27 (0.65–2.47)	0.93 (0.51–1.69)	0.85 (0.46–1.55)	0.93 (0.51–1.71)
≥46	1.81 (0.68–4.81)	1.86 (0.76–4.51)	0.83 (0.34–2.02)	1.27 (0.51–3.13)
**Sex**				
Male	ref	ref	ref	ref
Female	0.72 (0.52–0.99)	0.59 (0.44–0.79)	0.53 (0.39–0.70)	0.59 (0.44–0.80)
**Marital and parental status**				
Married with children	ref	ref	ref	ref
Married without children	1.74 (0.84–3.60)	0.88 (0.47–1.66)	1.13 (0.61–2.10)	1.08 (0.57–2.05)
Divorced/single	0.78 (0.49–1.24)	1.41 (0.91–2.19)	1.11 (0.72–1.70)	1.26 (0.81–1.96)
**Region**				
Fujian	ref	ref	ref	ref
Beijing	0.39 (0.24–0.63)	0.64 (0.40–1.02)	0.69 (0.43–1.12)	0.54 (0.34–0.87)
Sichuan	0.71 (0.44–1.15)	0.72 (0.45–1.15)	0.98 (0.62–1.54)	0.86 (0.54–1.37)
Shanxi	0.58 (0.33–1.03)	0.42 (0.24–0.75)	0.53 (0.30–0.92)	0.44 (0.22–0.68)
**Professional title**				
Resident	ref	ref	ref	ref
Attending physician	1.53 (0.89–2.63)	1.20 (0.72–1.98)	1.12 (0.68–1.84)	1.44 (0.87–2.39)
Associate chief physician	1.27 (0.62–2.57)	1.32 (0.67–2.59)	1.21 (0.62–2.37)	1.61 (0.82–3.17)
Chief physician	1.06 (0.42–2.65)	0.72 (0.30–1.74)	0.65 (0.27–1.55)	0.84 (0.35–2.03)
Other	0.96 (0.60–1.54)	0.69 (0.43–1.11)	0.74 (0.46–1.18)	0.68 (0.43–1.08)
**Years of practice**				
<5	ref	ref	ref	ref
5–10	0.82 (0.49–1.37)	1.43 (0.87–2.34)	1.19 (0.73–1.93)	0.94 (0.58–1.54)
11–15	1.03 (0.48–2.19)	1.38 (0.68–2.83)	0.74 (0.36–1.52)	0.89 (0.44–1.83)
16–20	0.54 (0.22–1.34)	0.92 (0.39–2.19)	1.11 (0.47–2.62)	0.79 (0.34–1.87)
>20	0.86 (0.29–2.53)	1.45 (0.53–3.99)	2.06 (0.75–5.67)	1.54 (0.55–4.28)
**Department type**				
High acuity emergency settings	ref	ref	ref	ref
Surgical procedural specialities	1.55 (0.78–3.11)	1.18 (0.62–2.27)	1.52 (0.79–2.94)	1.75 (0.91–3.38)
Complex chronic care	1.00 (0.52–1.93)	0.86 (0.46–1.61)	1.16 (0.61–2.18)	0.97 (0.52–1.82)
Mother-child health	0.97 (0.43–1.88)	0.84 (0.44–1.61)	1.03 (0.53–2.00)	1.04 (0.54–2.02)
Diagnostic supportive services	0.90 (0.43–1.88)	0.82 (0.40–1.66)	0.93 (0.46–1.91)	0.82 (0.40–1.67)
Other	1.31 (0.68–2.54)	0.87 (0.47–1.64)	1.22 (0.65–2.29)	1.08 (0.58–2.02)
**Weekly patient volume**				
<10	ref	ref	ref	ref
10–30	1.25 (0.79–1.98)	0.94 (0.61–1.46)	0.90 (0.58–1.38)	0.89 (0.58–1.39)
30–50	1.18 (0.68–2.04)	1.28 (0.76–2.14)	0.89 (0.54–1.48)	1.11 (0.66–1.87)
>50	0.79 (0.50–1.26)	1.10 (0.70–1.72)	0.78 (0.50–1.21)	0.89 (0.57–1.39)
**Night shift rotation**				
No night shifts	ref	ref	ref	ref
Required to work night shifts	1.61 (1.08–2.41)	1.91 (1.28–2.84)	2.03 (1.36–3.04)	2.09 (1.40–3.10)
**Income satisfaction**				
Dissatisfied	ref	ref	ref	ref
Neutral	0.63 (0.41–0.98)	0.41 (0.28–0.60)	0.79 (0.55–1.14)	0.40 (0.27–0.60)
Satisfied	0.24 (0.16–0.38)	0.22 (0.15–0.34)	0.42 (0.28–0.61)	0.20 (0.13–0.31)
**Research pressure**				
No/low pressure	ref	ref	ref	ref
High pressure	2.17 (1.42–3.31)	1.79 (1.25–2.57)	1.42 (0.99–2.20)	1.73 (1.19–2.51)
**Teaching pressure**				
No/low pressure	ref	ref	ref	ref
High pressure	2.29 (1.40–5.03)	1.66 (0.93–2.96)	2.18 (1.26–3.78)	1.78 (0.95–3.32)
**AI tool use**				
No	ref	ref	ref	ref
Yes	0.78 (0.57–1.07)	1.12 (0.84–1.50)	0.96 (0.72–1.27)	1.09 (0.81–1.45)

CI – confidence interval, MBI-HSS – Maslach Burnout Inventory—Human Services Survey, OR – odds ratio, PFI – Professional Fulfilment Index, ref – reference

Notably, research and teaching pressure showed different patterns across instruments. Research pressure was significantly associated with PFI burnout (OR = 2.17; 95% CI = 1.42–3.31), MBI emotional exhaustion (OR = 1.79; 95% CI = 1.25–2.57), and MBI composite burnout (OR = 1.73; 95% CI = 1.19–2.51). The association between research pressure and MBI depersonalisation was positive but not statistically significant (OR = 1.42; 95% CI = 0.99–2.20). In contrast, teaching pressure was significantly associated with PFI burnout (OR = 2.29; 95% CI = 1.40–5.03) and MBI depersonalisation (OR = 2.18; 95% CI = 1.26–3.78), but not with MBI emotional exhaustion (OR = 1.66; 95% CI = 0.93–2.96) or composite burnout (OR = 1.78; 95% CI = 0.95–3.32). This divergence may reflect differing mechanisms underlying these two occupational stressors. The use of AI tools was not associated with any burnout outcome across the instruments.

### Subgroup analyses by sex and professional title

To explore whether the effects of research and teaching pressure varied by gender and professional title, we conducted pre-specified subgroup analyses. In the overall sample, both research pressure (OR = 2.17; 95% CI = 1.42–3.31) and teaching pressure (OR = 2.29; 95% CI = 1.40–5.03) were significantly associated with increased burnout risk (Table S5 in the **Online Supplementary Document**). However, the magnitude of these associations varied substantially across subgroups. Research pressure significantly predicted burnout in both male and female physicians, with a stronger effect in males. Teaching pressure showed a positive but non-significant association in both sexes. When stratified by professional title, research pressure was strongly associated with burnout in junior physicians (*i.e.* residents, attending physicians, and other titles) (OR = 3.06; 95% CI = 1.73–5.42) but not in senior physicians (*i.e.* associate chief and chief physicians) (OR = 1.22; 95% CI = 0.58–2.58). Conversely, teaching pressure was significantly associated with burnout only in senior physicians (OR = 4.38; 95% CI = 1.29–14.89). We additionally conducted complete stratified models including all covariates (Table S6–8 in the **Online Supplementary Document**). Formal interaction tests did not provide statistically significant evidence that the associations of research pressure or teaching pressure with PFI-defined burnout differed by sex or professional title group (all *P* for interaction >0.071). Therefore, subgroup-specific estimates should be interpreted as exploratory rather than definitive evidence of subgroup differences. In exploratory sex-stratified models, the mother-child health category showed opposite directions of association with burnout among male and female physicians. These specialty-specific patterns were not part of the primary model conclusions and were interpreted as exploratory.

### Sensitivity analyses

We conducted a series of sensitivity analyses to assess the robustness of our primary findings (Table S9 in the **Online Supplementary Document**). First, treating PFI burnout score as a continuous outcome in linear regression yielded results consistent with the primary logistic models: teaching pressure, research pressure, income satisfaction, female sex, and night shift work remained independently associated with burnout, confirming that our findings are not artefacts of binary classification thresholds. Second, to examine whether our results were driven by the disproportionate representation of Fujian province, we repeated the analysis excluding Fujian physicians. In this geographically restricted subsample, research pressure remained a significant predictor of burnout (OR = 2.44; 95% CI = 1.24–4.79). However, teaching pressure was no longer significantly associated with burnout (OR = 1.34; 95% CI = 0.42–4.31), suggesting that the teaching-pressure association observed in the full sample may be context-specific and partly driven by the Fujian subsample rather than broadly generalisable. This regional heterogeneity underscores the importance of context-sensitive interpretations and interventions. Third, excluding physicians with ‘other’ professional titles to address potential heterogeneity in this category did not change any core findings.

## DISCUSSION

In this purposive multi-region institutional sample of 961 hospital physicians from four selected regions in China, 67.0% met PFI criteria for high burnout, 55% met MBI-HSS criteria for high burnout, 37.8% had moderate-to-severe depression, and 4.3% reported past-year suicidal ideation. The PFI and MBI-HSS showed only moderate agreement (Cohen’s kappa = 0.55), with the PFI classifying a substantially larger proportion of physicians as burned out. Male sex, night shift work, income dissatisfaction, and research pressure emerged as independent risk factors for burnout, while teaching pressure was associated with burnout in the full sample but showed less robustness in sensitivity analyses excluding Fujian participants. Notably, these stressors affected physicians differently: research pressure was more strongly associated with burnout in males and junior physicians, while teaching pressure predicted burnout only among senior physicians. In the broad six-category department classification, department type was not independently associated with burnout after adjustment. However, because these categories were conceptually defined and internally heterogeneous, particularly the ‘other specialities’ group, this finding should not be interpreted as evidence that speciality-specific differences are absent.

Both the assessed PFI (67.0%) and MBI-HSS (55.0%) burnout rates are consistent with the standards of Chinese academic medicine. International benchmarks vary widely across instruments and populations [3]. Among the US physicians, Shanafelt *et al.* reported an MBI-based prevalence of 54.4% in 2015, up from 45.5% in 2011 [1]. Among Chinese physicians, a 2022 meta-analysis by Zheng *et al.* encompassing 64 studies and 48,638 participants reported a pooled MBI-HSS burnout rate of 67.3% (95% CI = 51.6–81.2) [30], which exceeds the 55.0% MBI-HSS rate we observed – a discrepancy consistent with a reported post-2018 decline in Chinese physician burnout prevalence and with our purposive rather than our nationally representative sampling frame. These comparisons provide contextual benchmarks only because our sample was purposively selected and heavily weighted toward Fujian Province; the observed prevalence estimates should not be interpreted as nationally representative.

The moderate inter-instrument agreement highlights methodological challenges in cross-national burnout comparisons. The PFI cut-off of ≥1.33 was established in the US physician sample and may not be optimally calibrated for Chinese populations. The observed discordance may partly reflect differences in construct definition and response format: the PFI treats burnout as a unified construct combining work exhaustion and interpersonal disengagement, whereas the MBI-HSS assesses separate dimensions using frequency-based response options [31]. The original PFI validation study also reported a sensitivity of 72% and specificity of 84% against MBI-defined burnout, confirming that non-identical classification is an expected psychometric property of the instrument itself [16]. Although East Asian-Western comparisons have reported cultural response tendencies in rating scales [32], we did not directly measure response style, acquiescence bias, or cultural reporting patterns; therefore, we cannot determine whether these factors contributed to the observed instrument discordance. This suggests that the absolute prevalence of burnout may be sensitive to the cut-off selected. If the PFI threshold is too low for this population, burnout prevalence may be overestimated; if Chinese physicians interpret or report burnout-related items differently, the same threshold could also underestimate clinically meaningful distress. Therefore, the PFI estimate should be read as an instrument-defined classification rather than a culturally calibrated prevalence estimate. Despite these uncertainties in prevalence estimates, the pattern of risk factor associations was highly consistent across both instruments, lending convergent validity to our core findings and suggesting that moderate concordance reflects measurement-level divergence rather than assessment of fundamentally different constructs.

The 162 physicians burned out by PFI but not MBI-HSS are a clinically important subgroup. One possible explanation is that some of these individuals may have earlier or less severe burnout presentations that are captured by the PFI’s unified construct but do not meet MBI-HSS dimensional thresholds [16,32]. This pattern may reflect differences in how the two instruments classify burnout, including the PFI’s unified cut-off and the MBI-HSS’s reliance on dimension-specific thresholds. It may also indicate that the PFI captures earlier or broader manifestations of burnout, although longitudinal data would be needed to test this interpretation. Either interpretation has practical significance: prevalence estimates are instrument-dependent, and relying on a single tool risks systematically misclassifying physicians who may benefit most from early intervention.

Although measured using single binary self-report items, perceived research pressure and perceived teaching pressure showed different association patterns across burnout dimensions. Research pressure was consistently associated with PFI burnout, MBI emotional exhaustion, and MBI composite burnout. Teaching pressure, by contrast, was significantly associated only with PFI burnout and MBI depersonalisation. This divergence aligns with the Job Demands-Resources framework and suggests distinct role-related pathways for specific occupational demands [26], although we did not test them directly. Research pressure – the push for publications, grants, and academic recognition – appears to function as a chronic, generalised demand that progressively depletes emotional reserves. Although evidence from Chinese university faculty cannot be directly equated with hospital physicians, it provides relevant contextual support: academic research stress has been associated with occupational burnout, particularly emotional exhaustion, in Chinese academic settings [9]. This pattern aligns with broader evidence that the publish-or-perish culture in academic medicine is a principal organisational driver of emotional exhaustion among physicians [17]. For teaching pressure, the observed association may reflect a more context-specific pathway. When physicians feel overwhelmed by teaching responsibilities – often added to full clinical loads without protected time or institutional recognition – they may cope by distancing themselves emotionally from those they supervise. Consistent with this interpretation, large-scale studies of physician burnout have identified time pressure and poor job control as the strongest workplace predictors of depersonalisation, dimensions directly implicated when teaching duties are imposed without adequate autonomy or support [33]. Academic physicians are particularly vulnerable, given systematic under-compensation for teaching and the progressive erosion of protected teaching time as clinical demands expand [34].

The differential vulnerability observed across subgroups reflects the structural realities of Chinese academic medicine at different career stages. Research pressure was more strongly associated with burnout among male and junior physicians, consistent with a system in which publication output and grant acquisition serve as the primary criteria for early-career promotion [7,35]. Junior physicians face a compressed timeline in which clinical training, research productivity, and career advancement compete simultaneously for cognitive and emotional resources [34,35]. Teaching pressure, by contrast, predicted burnout only among senior physicians – a pattern consistent with a career-stage shift in which supervisory and mentorship responsibilities accumulate without corresponding protected time or recognition in formal performance metrics [34]. Because formal interaction tests did not support definitive effect modification by sex or professional title group for research or teaching pressure (Table S8 in the **Online Supplementary Document**), these subgroup findings should be interpreted as exploratory.

The regional disparity regarding teaching pressure likely stems from local structural factors. Fujian's dense concentration of medical schools and teaching hospitals may create a particularly high teaching burden that amplifies this association locally [7]. Research pressure, by contrast, remained significant across all regional subsamples, suggesting a more uniform national mechanism. This distinction may inform future intervention design: strategies targeting teaching-related burnout may require local tailoring, whereas research pressure may be a more broadly relevant target for future institutional evaluation.

The finding that male sex was associated with higher burnout runs counter to most international evidence, in which female physicians have been consistently identified as bearing higher burnout risk [7,36]. The observed sex difference may reflect China-specific professional and social contexts, although this study did not directly measure these mechanisms. A nationwide survey of Chinese medical staff found that male sex was an independent risk factor for burnout after multivariable adjustment [7]. Studies of Chinese healthcare workers during the COVID-19 pandemic further showed that burnout rates were significantly higher in male than female staff [37]. One possible explanation is that publication-based career benchmarks may carry particular salience for some male physicians in the Chinese academic medical system, but this interpretation remains theoretical because publication pressure by sex was not directly measured [17,38].

Although department type was not independently associated with burnout in the primary adjusted model, exploratory sex-stratified analyses suggested a descriptive speciality-specific pattern in the mother-child health category, where male physicians showed lower burnout risk. In comparison, female physicians showed higher burnout risk relative to other departments. One possible explanation is that female physicians in maternal and child health settings may face disproportionate emotional demands: patients may expect greater empathy and emotional support from female physicians, yet institutional resources may not always accommodate these expectations [39]. In a speciality defined by intimate, high-stakes care for mothers and newborns – where patients are particularly likely to expect emotional attunement and extended communication from female physicians – this mismatch between patient expectations and allocated time may be especially pronounced [40]. However, because we did not directly measure patient expectations and emotional workload, this interpretation should be regarded as hypothesis-generating.

Night shift work and income satisfaction emerged as significant predictors of physician well-being. The association between night shifts and burnout is consistent with extensive prior evidence linking overnight work to emotional exhaustion through disrupted sleep, sustained vigilance demands, and reduced recovery time [41]. Income satisfaction, by contrast, functioned as a protective factor against both burnout and depressive symptoms. This finding aligns with effort-reward imbalance theory, which posits that perceived discrepancy between occupational demands and material rewards is a primary driver of chronic work-related stress [35].

Depression findings merit brief consideration alongside burnout. Although teaching pressure and income dissatisfaction predicted both outcomes, two factors showed divergent patterns. Research pressure was robustly associated with burnout but showed only a non-significant trend for depression, consistent with evidence that burnout represents an occupation-specific exhaustion syndrome distinct from the broader cognitive-affective dysregulation of depressive illness [42]. Night shift work similarly predicted burnout but not depression, likely reflecting that circadian disruption depletes emotional resources through acute fatigue rather than the sustained ruminative processes implicated in depression [43]. The stronger protective effect of income satisfaction against burnout than depression aligns with effort-reward imbalance theory, under which salary dissatisfaction most directly fuels occupation-specific distress [44]. These patterns suggest that burnout and depression, though frequently comorbid (32.7%), respond to partially distinct occupational pathways.

While recent literature on generative AI in Chinese healthcare emphasises clinical efficiency, its occupational impact on practitioners remains largely unexamined. The null finding could reflect the limited granularity of our dichotomous AI-use measure, reverse causation, or simply that current generative AI tools do not yet meaningfully reduce physician workload. Future studies should incorporate longitudinal designs and objective workload measures, and more detailed assessment of AI use, including frequency, purpose, competency, and workflow integration, to clarify whether generative AI adoption may confer measurable benefits for physician well-being.

Exploratory analyses of suicidal ideation must be interpreted with caution given the small number of cases. In the reduced model, higher teaching pressure remained positively associated with suicidal ideation, consistent with previous evidence linking academic performance demands to psychological crisis among Chinese physicians [8,45]. However, this finding should be regarded as hypothesis-generating and requires replication in larger samples before drawing causal or predictive inferences.

The findings of this study carry implications beyond China’s healthcare system. The moderate instrument concordance observed reinforces a global methodological concern: burnout prevalence estimates derived from Western-normed cut-offs risk systematic misclassification when applied to non-Western populations. The divergent roles of research and teaching pressure further reflect a structural tension between clinical service, academic output, and educational responsibility that is increasingly common in academic medical centres across middle-income countries undergoing rapid research capacity expansion. Finally, income satisfaction as the strongest modifiable protective factor highlights an effort-reward imbalance structurally embedded in public hospital systems globally, particularly where physician salaries have not kept pace with rising occupational demands. These patterns suggest that effective burnout prevention in non-Western academic medical systems requires culturally adapted instruments, career-stage-specific interventions, and salary structures that adequately reflect occupational demands.

### Strengths and limitations

This study has several limitations. First, the cross-sectional design precludes causal inference; prospective studies are needed to establish whether identified risk factors precede or follow the onset of burnout and depression. Second, all measures were self-reported, introducing the possibility of social desirability bias. Third, although the four regions were selected to capture variation in geography and economic development, institutional access and existing collaborations constrained site selection. Therefore, the sample may not fully represent the diversity of Chinese hospital practice environments. In addition, the disproportionate concentration of participants from Fujian Province (64.4%) further limits the generalisability of prevalence estimates. The attenuation of the teaching pressure–burnout association after excluding Fujian participants is best understood as evidence of regional heterogeneity rather than instability of the overall findings and warrants confirmation in nationally representative samples. Fourth, we based department classification on conceptual work-demand profiles rather than empirically derived clusters. Substantial heterogeneity may remain within categories, particularly the broad ‘other specialities’ group, which may have obscured speciality-specific associations. Fifth, we scored the PROMIS Depression Short Form using T-score calibrations normed on a United States general population, and no published study has established criterion-validated cut-offs for Chinese adults; cultural differences in depressive symptom expression may therefore systematically underestimate true depression burden. Similarly, the PFI cut-off of ≥1.33 was derived from the US physician samples and has not been criterion-validated among Chinese physicians. Applying this cut-off may have introduced threshold-related misclassification and influenced the estimated burnout prevalence. Therefore, PFI-based prevalence should be interpreted as an instrument-defined classification rather than a culturally calibrated prevalence estimate. In addition, although response style may influence cross-cultural scale performance, this study did not directly measure response style, acquiescence bias, or cultural reporting patterns. Hence, explanations for PFI-MBI discordance remain inferential. Sixth, we measured both research pressure and teaching pressure using single binary self-report items. Although this approach captures perceived domain-specific academic pressure, these constructs are multidimensional and may vary by intensity, frequency, duration, institutional expectations, protected time, promotion requirements, and perceived burden. This measurement approach may have introduced measurement error and limited our ability to distinguish which specific components of research and teaching responsibilities were most strongly associated with burnout and depression. Seventh, although the overall sample size was sufficient for prevalence estimation and modelling common outcomes, the study was not powered for subgroup analyses or rare outcomes such as suicidal ideation. Given only 41 cases of suicidal ideation, we fitted a reduced model to limit overfitting. However, estimates from this model and subgroup findings should still be interpreted as exploratory because of wide confidence intervals, smaller stratum-specific sample sizes, and potential statistical instability. Finally, the null association observed for generative AI tool use should be interpreted with caution, as this variable may not adequately capture the frequency, purpose, competency, or degree of AI integration into clinical workflows.

Future research should prioritise prospective cohort designs to establish causal pathways between occupational demands and physician mental health outcomes, and cultural validation studies to derive PFI cut-offs calibrated to Chinese populations through receiver operating characteristic analysis. Qualitative inquiry into the lived experiences of physicians with discordant burnout classifications, and international comparative studies examining whether the sex reversal in burnout risk observed here is specific to Chinese academic medicine, would further advance both measurement science and intervention development in this field.

## CONCLUSIONS

We found a high burden of burnout and depression among participating hospital physicians in a purposive multi-region sample from China, with burnout affecting two-thirds, depression affecting more than one-third, and suicidal ideation affecting more than 4% of doctors. The modest concordance between burnout instruments highlights the need for culturally adapted assessment tools and caution in applying Western cut-off scores to Chinese populations. Research pressure emerged as a more consistent cross-regional correlate of burnout. Teaching pressure was associated with burnout in the full sample, but this association was not retained after excluding Fujian province, suggesting that teaching-related burnout may be more context-specific. Income satisfaction was the strongest modifiable protective correlate identified, suggesting that compensation-related factors may warrant further evaluation in future longitudinal or interventional research. The substantial burden of mental health challenges among these physicians underscores the need for targeted structural reforms in hospital management. Future longitudinal and intervention studies are needed to determine whether modifying academic workload, teaching support, promotion criteria, or effort-reward structures can improve physician mental health.

## Additional material


Online Supplementary Document

## Data Availability

**Data availability**: The data used and analysed are available from the corresponding author upon reasonable request.
