# Supplementary material for: Burnout, depression, and suicidal ideation among Chinese hospital physicians: prevalence and the distinct roles of research and teaching pressure
Source: J Glob Health. 2026 Oct 23;16:04323. doi: 10.7189/jogh.16.04323 (PMC13693296; doi:10.7189/jogh.16.04323)
Supplement: Supplementary file 1 [file jogh-16-04323-s001.pdf]

Supplement to: Bao X, Zhao Y, Yang T, Huang P, Guo D. Burnout, depression, and suicidal ideation among Chinese hospital physicians: prevalence and the distinct roles of research and teaching pressure. J Glob Health. 2026;16:04323.

Table S1. Regional Economic Characteristics and Sample Distribution Across Multiple Provinces in China

Region	City/District	Sample Size (n)	Percentage (%)	GDP per Capita (¥, 2023)	Economic Level
Fujian	Ningde	619	64.4	120,619	High
Sichuan	Luzhou	127	13.2	63,913	Moderate
Beijing	Fengtai	118	12.3	200,278	Very High
Shanxi	Taiyuan	97	10.1	102,922[a]	Moderate-High
Total		961	100.0		

[a] Most recent available data (2022).
Economic development data were obtained from the National Bureau of Statistics of China and respective municipal statistical bureaus.
Economic level categorization: Very High (GDP per capita >180% of national average ¥89,358 in 2023), High (120–180%), Moderate-High (100–120%), Moderate (60–100%).

Table S2. Burnout Prevalence by Department Type Among Chinese Hospital Physicians (N = 961)

Department Type	Total n (%)	High Burnout (PFI ≥1.33) n (%)	Low/No Burnout n (%)	P Value
Overall	961 (100%)	644 (67.0%)	317 (33.0%)	0.228
High Acuity / Emergency Settings	59 (6.1%)	41 (69.5%)	18 (30.5%)	0.676
Surgical / Procedural Specialties	173 (18.0%)	115 (66.5%)	58 (33.5%)	0.868
Surgery	115 (66.5%)	79 (68.7%)	36 (31.3%)	0.683
Orthopedics	58 (33.5%)	36 (62.1%)	22 (37.9%)	0.409
Complex Chronic Care	218 (22.7%)	162 (74.3%)	56 (25.7%)	0.009
Internal Medicine	210 (96.3%)	156 (74.3%)	54(25.7%)	0.264
Mother-Child Health	171 (17.8%)	105 (61.4%)	66 (38.6%)	0.085
Obstetrics & Gynecology	91 (53.2%)	50(54.9%)	41 (45.1%)	0.010
Diagnostic / Supportive Services	101 (10.5%)	73 (72.3%)	28 (27.7%)	0.234
Imaging/Radiology	90 (89.1%)	66 (73.3%)	24(26.7%)	0180
Other Departments	239 (24.9%)	148 (61.9%)	91 (38.1%)	0.054

Burnout defined as Professional Fulfillment Index (PFI) mean score ≥1.33 on a 0–4 scale. Subcategory percentages for Surgery, Orthopedics, Internal Medicine, Obstetrics & Gynecology, and Imaging/Radiology are expressed as the proportion within their parent category. P values for department categories were derived from chi-square tests comparing burnout prevalence within each subcategory to the overall sample.

Only departments with more than 50 physicians are included.

Table S3. Adjusted Associations Between Risk Factors and Mental Health Outcomes Among Chinese Physicians[a]

Risk factor	High burnout OR (95% CI)	Moderate-severe depression OR (95% CI)	Suicidal ideation OR (95% CI)
**Demographic factors**			
**Age group**			
≤ 35 years	1.00	1.00	1.00
36 – 45 years	1.27 (0.65 – 2.47)	1.14 (0.62 – 2.09)	0.34 (0.09 – 1.35)
≥ 46 years	1.81 (0.68 – 4.81)	1.18 (0.49 – 2.82)	0.11 (0.01 – 0.79)
**Sex**			
Male	1.00	1.00	1.00
Female	0.72 (0.52 – 0.99)	0.62(0.46 – 0.83)	0.65 (0.32 – 1.34)
**Marital & Parental status**			
Married with children	1.00	1.00	1.00
Married without children	1.74 (0.84 – 3.60)	0.97 (0.52 – 1.82)	0.29 (0.03 – 2.49)
Divorced/Single	0.78 (0.49 – 1.24)	1.03 (0.66 – 1.60)	0.82 (0.29 – 2.33)
**Region**			
Fujian	1.00	1.00	1.00
Beijing	0.39 (0.24 – 0.63)	0.76 (0.47 – 1.22)	0.47 (0.10 – 2.20)
Sichuan	0.71 (0.44 – 1.15)	0.77 (0.48 – 1.22)	1.82 (0.67 – 4.96)
Shanxi	0.58 (0.33 – 1.03)	0.52 (0.29 – 0.92)	0.32 (0.06 – 1.70)
**Professional factors**			
**Professional title**			
Resident	1.00	1.00	1.00
Attending physician	1.53 (0.89 – 2.63)	1.35 (0.82 – 2.23)	3.14 (0.90 – 10.89)
Associate chief physician	1.27 (0.62 – 2.57)	0.97 (0.50 – 1.89)	1.31 (0.26 – 6.72)
Chief physician	1.06 (0.42 – 2.65)	0.61 (0.26 – 1.46)	2.07 (0.27 – 15.89)
Other	0.96 (0.60 – 1.54)	0.59 (0.36 – 0.97)	1.64 (0.50 – 5.43)
**Years of practice**			
≤5 years	1.00	1.00	1.00
6 – 10 years	0.82 (0.49 – 1.37)	1.07 (0.65 – 1.76)	0.65 (0.19 – 2.22)
11 – 15 years	1.03 (0.48 – 2.19)	0.90 (0.44 – 1.88)	1.33 (0.27 – 6.67)
16 – 20 years	0.54 (0.22 – 1.34)	1.02 (0.43 – 2.41)	4.75 (0.67 – 33.79)
> 20 years	0.86 (0.29 – 2.53）	1.23 (0.45 – 3.36)	5.85 (0.59 – 58.50)
**Department**			
**Department type**			

Risk factor	High burnout<br>OR (95% CI)	Moderate-severe depression<br>OR (95% CI)	Suicidal ideation<br>OR (95% CI)
High acuity/emergency settings	1.00	1.00	1.00
Surgical/procedural specialties	1.55 (0.78 – 3.11)	0.91 (0.48 – 1.73)	0.47 (0.10 – 2.15)
Complex chronic care	1.00 (0.52 – 1.93)	0.77 (0.41 – 1.43)	0.70 (0.17 – 2.86)
Mother-child health	0.97 (0.49 – 1.93)	0.62 (0.32 – 1.18)	0.54 (0.12 – 2.41)
Diagnostic supportive services	0.90 (0.43 – 1.88)	0.45 (0.22 – 0.92)	0.49 (0.09 – 2.73)
Other	1.31 (0.68 – 2.54)	0.73 (0.39 – 1.35)	1.01 (0.26 – 3.90)
**Work-related factors**			
**Weekly patient volume**			
< 10 patients	1.00	1.00	1.00
10 – 30 patients	1.25 (0.78 – 1.98)	0.99 (0.64 – 1.54)	0.62 (0.23 – 1.70)
30 – 50 patients	1.18 (0.68 – 2.04)	1.02 (0.61 – 1.70)	0.32 (0.09 – 1.10)
> 50 patients	0.79 (0.50 – 1.26)	0.95 (0.60 – 1.49)	0.55 (0.20 – 1.51)
**Night shift rotation**			
No night shifts	1.00	1.00	1.00
Required to work night shifts	1.61 (1.08 – 2.41)	1.37 (0.92 – 2.05)	2.44 (0.76 – 7.79)
**Income satisfaction**			
Dissatisfied	1.00	1.00	1.00
Neutral	0.63 (0.41 – 0.98)	0.64 (0.44 – 0.92)	0.64 (0.28 – 1.44)
Satisfied	0.24 (0.16 – 0.38)	0.37 (0.25 – 0.55)	0.50 (0.21 – 1.19)
**Research pressure**			
No/Low pressure	1.00	1.00	1.00
High pressure	2.17 (1.42 – 3.31)	1.33 (0.93 – 1.89)	1.03 (0.45 – 2.37)
**Teaching pressure**			
No/Low pressure	1.00	1.00	1.00
High pressure	2.29 (1.40 – 5.03)	2.06 (1.21 – 3.52)	3.72 (1.48 – 9.33)
**Technology factor**			
**AI tool use**			
No	1.00	1.00	1.00
Yes	0.78 (0.57 – 1.07)	0.86 (0.65 – 1.14)	0.88 (0.45 – 1.72)

Abbreviations: aOR, adjusted odds ratio; AI, artificial intelligence; CI, confidence interval; PFI, Professional Fulfillment Index; SD, standard deviation.

[a] All models adjusted for all variables shown in table. Sample size: n=961 for all models. Models exclude work hours and patient volume variables due to missing data and collinearity with night shift variable.

Table S4. Reduced Logistic Regression Model for Suicidal Ideation Among Chinese Hospital Physicians

Risk factor	Category	Adjusted OR	95% CI	*P* value
Age group	<=35 years	1.00	Reference	
Age group	36–45 years	1.18	0.58–2.39	0.648
Age group	>=46 years	0.70	0.25–1.95	0.495
Sex	Male	1.00	Reference	
Sex	Female	0.60	0.31–1.15	0.127
Income satisfaction	Dissatisfied	1.00	Reference	
Income satisfaction	Neutral	0.80	0.37–1.74	0.579
Income satisfaction	Satisfied	0.60	0.26–1.39	0.235
Research pressure	No/Low pressure	1.00	Reference	
Research pressure	High pressure	0.93	0.42–2.07	0.863
Teaching pressure	No/Low pressure	1.00	Reference	
Teaching pressure	High pressure	3.61	1.50–8.71	0.004

Abbreviations: OR, odds ratio; CI, confidence interval.
The outcome was suicidal ideation in the past 12 months. The reduced logistic regression model included age group, sex, income satisfaction, research pressure, and teaching pressure. The analytic sample included 961 physicians, of whom 41 reported suicidal ideation. High research and teaching pressure were defined as the highest response category for the respective pressure items. Results should be interpreted as exploratory because of the small number of suicidal ideation events.

Table S5. Subgroup Analyses: Associations of Research and Teaching Pressure with Burnout (PFI) by Gender and Professional Title [a]

Subgroup	Research Pressure (High vs. Low) OR (95% CI) for Burnout	Teaching Pressure (High vs. Low) OR (95% CI) for Burnout
**Overall (N = 961)**	2.17 (1.42–3.31)	2.29 (1.40–5.03)
**Sex**		
Male (n = 464)	2.88 (1.46–5.71)	2.19 (0.68–7.05)
Female (n = 497)	2.10 (1.17–3.76)	2.63 (0.87–7.96)
**Professional Title**		
Junior Physicians [a] (n = 678)	3.06 (1.73–5.42)	2.31 (0.73–7.30)
Senior Physicians [b] (n = 283)	1.22 (0.58–2.58)	4.38 (1.29–14.89)

Abbreviations: aOR, adjusted odds ratio; CI, confidence interval; PFI, Professional Fulfillment Index.
a. Junior physicians: residents, attending physicians, and "other" titles.
b. Senior physicians: associate chief physicians and chief physicians.
The outcome is burnout (PFI ≥1.33) in all models. Complete stratified analyses including all covariates are presented in Table S6 (gender) and S7 (professional title).

Table S6. Sex-Stratified Logistic Regression of Burnout (PFI) Among Chinese Hospital Physicians

Risk Factor	Male Physicians (n = 464) OR (95% CI)	Female Physicians (n = 497) OR (95% CI)
Demographic Factors		
Age Group		
≤35 years	1.00	1.00
36–45 years	1.48 (0.51–4.24)	1.14 (0.46–2.85)
≥46 years	1.59 (0.34–7.38)	1.83 (0.48–7.00)
Marital & Parental Status		
Married with children	1.00	1.00
Married without children	1.42 (0.44–4.59)	2.16 (0.79–5.93)
Divorced/Single	0.50 (0.22–1.15)	1.03 (0.56–1.87)
Region		
Fujian	1.00	1.00
Beijing	0.19 (0.08–0.46)	0.54 (0.29–0.99)
Sichuan	1.55 (0.65–3.70)	0.55 (0.29–1.03)
Shanxi	0.36 (0.14–0.98)	0.63 (0.29–1.35)
Professional Factors		
Professional Title		
Resident	1.00	1.00
Attending physician	1.48 (0.59–3.70)	1.39 (0.67–2.87)
Associate chief physician	1.90 (0.53–6.77)	0.75 (0.30–1.87)
Chief physician	1.57 (0.35–7.08)	0.61 (0.16–2.38)
Other	1.10 (0.48–2.51)	0.93 (0.51–1.70)
Years of Practice		
≤5 years	1.00	1.00
6–10 years	0.49 (0.21–1.17)	1.20 (0.61–2.38)
11–15 years	0.89 (0.24–3.33)	1.34 (0.50–3.60)
16–20 years	0.52 (0.11–2.51)	0.66 (0.20–2.16)
>20 years	0.95 (0.15–6.19)	1.07 (0.25–4.53)
Department Type		
High Acuity / Emergency Settings	1.00	1.00
Surgical/Procedural Specialties	0.65 (0.17–2.44)	2.38 (0.95–5.95)
Complex Chronic Care	0.42 (0.12–1.49)	1.41 (0.59–3.34)
Mother-Child Health	0.21 (0.06–0.79)	2.61 (1.05–6.50)
Diagnostic/Supportive Services	0.61 (0.15–2.45)	1.00 (0.39–2.61)
Other Departments	0.48 (0.13–1.75)	2.11 (0.89–4.99)
Work–Related Factors		
Weekly Patient Volume		
<10 patients	1.00	1.00
10–30 patients	0.91 (0.42–1.99)	1.66 (0.88–3.11)
30–50 patients	0.60 (0.24–1.52)	1.80 (0.86–3.74)
>50 patients	0.33 (0.15–0.75)	1.27 (0.70–2.32)
Night Shift Rotation		
No night shifts	1.00	1.00
Required to work night shifts	2.08 (1.09–3.95)	1.49 (0.86–2.60)

Risk Factor	Male Physicians (n = 464)<br>OR (95% CI)	Female Physicians (n = 497)<br>OR (95% CI)
Income Satisfaction		
Dissatisfied	1.00	1.00
Neutral	0.98 (0.50–1.95)	0.43 (0.23–0.81)
Satisfied	0.30 (0.16–0.58)	0.18 (0.10–0.36)
Research Pressure		
No/Low pressure	1.00	1.00
High pressure	2.88 (1.46–5.71)	2.10 (1.17–3.76)
Teaching Pressure		
No/Low pressure	1.00	1.00
High pressure	2.19 (0.68–7.05)	2.63 (0.87–7.96)
Technology Factor		
AI Tool Use		
No	1.00	1.00
Yes	0.67 (0.41–1.09)	0.92 (0.60–1.42)

OR = odds ratio; CI = 95% confidence interval; PFI = Professional Fulfillment Index. Male n = 464 (48.3%); Female n = 497 (51.7%). Burnout defined as PFI mean score ≥1.33 on a 0–4 scale.

Table S7. Stratified Logistic Regression of Burnout (PFI) by Professional Title Group Among Chinese Hospital Physicians

Risk Factor	Junior Physicians (Resident + Attending + Other) n = 678<br>OR (95% CI)	Senior Physicians (Associate Chief + Chief) n = 283<br>OR (95% CI)
Demographic Factors		
Age group		
≤35 years	Reference	Reference
36–45 years	1.66 (0.81–3.37)	6.53 (0.30–140.1)
≥46 years	0.58 (0.13–2.68)	19.23 (0.77–481.7)
Sex		
Male	Reference	Reference
Female	0.81 (0.56–1.18)	0.60 (0.32–1.13)
Marital & Parental Status		
Married with children	Reference	Reference
Married without children	1.89 (0.89–4.03)	—
Divorced/Single	0.98 (0.58–1.64)	0.21 (0.07–0.66)
Region		

Risk Factor	Junior Physicians (Resident + Attending + Other) n = 678<br>OR (95% CI)	Senior Physicians (Associate Chief + Chief) n = 283<br>OR (95% CI)
Fujian	Reference	Reference
Beijing	0.56 (0.28–1.12)	0.31 (0.15–0.64)
Sichuan	0.66 (0.37–1.19)	1.30 (0.50–3.37)
Shanxi	0.51 (0.28–0.93)	—
Professional Factors		
Years of Practice		
≤5 years	Reference	Reference
6–10 years	0.98 (0.59–1.63)	—
11–15 years	1.55 (0.71–3.40)	—
16–20 years	0.36 (0.12–1.07)	—
>20 years	2.92 (0.52–16.56)	—
Department Type		
High Acuity / Emergency Settings	Reference	Reference
Surgical/Procedural	1.34 (0.59–3.03)	2.64 (0.55–12.74)
Complex Chronic Care	0.93 (0.43–2.00)	1.01 (0.22–4.74)
Mother-Child Health	0.88 (0.38–2.03)	1.38 (0.30–6.37)
Diagnostic/Supportive	0.66 (0.29–1.50)	4.89 (0.51–47.41)
Other	1.32 (0.61–2.86)	1.21 (0.27–5.52)
Work－Related Factors		
Weekly Patient Volume		
<10 patients	Reference	Reference
10–30 patients	1.40 (0.85–2.29)	1.01 (0.09–10.92)
30–50 patients	1.46 (0.76–2.80)	0.76 (0.07–8.13)
>50 patients	0.76 (0.46–1.26)	0.83 (0.08–8.56)
Night Shift Rotation		
No night shifts	Reference	Reference

Risk Factor	Junior Physicians (Resident + Attending + Other) n = 678<br>OR (95% CI)	Senior Physicians (Associate Chief + Chief) n = 283<br>OR (95% CI)
Required to work	1.77 (1.03–3.04)	2.29 (1.16–4.52)
Income Satisfaction		
Dissatisfied	Reference	Reference
Neutral	0.76 (0.47–1.25)	0.45 (0.14–1.37)
Satisfied	0.25 (0.15–0.42)	0.24 (0.08–0.71)
Research Pressure		
No/Low pressure	Reference	Reference
High pressure	3.06 (1.73–5.42)	1.22 (0.58–2.58)
Teaching Pressure		
No/Low pressure	Reference	Reference
High pressure	2.31 (0.73–7.30)	4.38 (1.29–14.89)
Technology Factor		
AI Tool Use		
No	Reference	Reference
Yes	0.78 (0.53–1.14)	0.62 (0.34–1.15)

OR = odds ratio; CI = 95% confidence interval; PFI = Professional Fulfillment Index. Burnout defined as PFI mean score ≥1.33 on a 0–4 scale.

Table S8. Formal Interaction Tests for Subgroup Differences in PFI − Defined Burnout

Interaction tested	Outcome	Interaction aOR (95% CI)	P for interaction
Research pressure × Sex	PFI high burnout	0.88 (0.39–1.98)	0.762
Teaching pressure × Sex	PFI high burnout	1.02 (0.22–4.65)	0.977
Research pressure × Professional title	PFI high burnout	0.46 (0.20–1.07)	0.071
Teaching pressure × Professional title	PFI high burnout	1.00 (0.22–4.59)	0.996

aOR = adjusted odds ratio; CI = confidence interval; PFI = Professional Fulfillment Index. Each interaction term was tested in a separate multivariable logistic regression model adjusted for the same covariates as the primary burnout model. Professional title group was coded as junior versus senior physicians

Table S9. Sensitivity Analyses: Adjusted Associations Between All Risk Factors and Mental Health Outcomes Under Four Alternative Model Specifications (N = 961)

Risk factor	PFI (Primary Analysis)	SA1: PFI Continuous	SA2: Excluding Fujian Province (n = 342)	SA3: Excluding 'Other' Title (n = 814)
	Burnout OR (95% CI)	Score β (95% CI)	Burnout OR (95% CI)	Burnout OR (95% CI)
Demographic factors				
Age group				
≤ 35 years	1.00		1.00	1.00
36–45 years	1.27 (0.65–2.47)		0.78 (0.24–2.55)	1.13 (0.54–2.35)
≥ 46 years	1.81 (0.68–4.81)		1.15 (0.25–5.33)	1.66 (0.59–4.63)
β per age group increase		–0.43 (–1.75 to 0.90)		
Sex				
Male	1.00		1.00	1.00
Female	0.72 (0.52–0.99)	–2.25 (–3.19 to –1.30)	0.66 (0.39–1.14)	0.73 (0.52–1.03)
Marital & parental status				
Married with children	1.00		1.00	1.00
Married without children	1.74 (0.84–3.60)		2.85 (0.80–10.22)	1.63 (0.74–3.58)
Divorced/Single	0.78 (0.49–1.24)		0.93 (0.39–2.18)	0.75 (0.45–1.28)
β per unit increase		–0.46 (–1.11 to 0.20)		
Region				
Fujian	1.00			1.00
Beijing	0.39 (0.24–0.63)		1.00	0.40 (0.24–0.65)
Sichuan	0.71 (0.44–1.15)		1.50 (0.76–2.96)	0.74 (0.43–1.27)
Shanxi	0.58 (0.33–1.03)		1.78 (0.68–4.65)	0.49 (0.25–0.95)
β per unit change		–0.42 (–0.91 to 0.06)		
Professional factors				
Professional title				
Resident	1.00	–0.29 (–0.65 to 0.07)	1.00	1.00
Attending physician	1.53 (0.89–2.63)		1.96 (0.72–5.33)	1.51 (0.82–2.78)
Associate chief physician	1.27 (0.62–2.57)		1.00 (0.31–3.30)	1.25 (0.54–2.88)
Chief physician	1.06 (0.42–2.65)		0.65 (0.14–3.02)	1.06 (0.38–3.00)
Other	0.96 (0.60–1.54)		1.18 (0.52–2.66)	—
β per title level increase		–0.29 (–0.65 to 0.07)		
Years of practice				
< 5 years	1.00		1.00	1.00
6–10 years	0.82 (0.49–1.37)		1.19 (0.44–3.23)	0.93 (0.51–1.71)
11–15 years	1.03 (0.48–2.19)		1.86 (0.46–7.49)	1.04 (0.43–2.50)

Risk factor	PFI (Primary Analysis) Burnout OR (95% CI)	SA1: PFI Continuous Score β (95% CI)	SA2: Excluding Fujian Province (n = 342) Burnout OR (95% CI)	SA3: Excluding 'Other' Title (n = 814) Burnout OR (95% CI)
16–20 years	0.54 (0.22–1.34)		1.42 (0.29–6.84)	0.69 (0.25–1.95)
> 20 years	0.86 (0.29–2.53)		2.27 (0.39–13.27)	0.97 (0.29–3.24)
β per 5–year increase		0.67 (–0.07 to 1.41)		
Department				
Department type				
High acuity emergency settings	1.00		1.00	1.00
Surgical procedural specialties	1.55 (0.78–3.11)		1.93 (0.60–6.17)	1.25 (0.56–2.77)
Complex chronic care	1.00 (0.52–1.93)		1.00 (0.35–2.90)	0.69 (0.32–1.47)
Mother child health	0.97 (0.43–1.88)		1.59 (0.48–5.25)	0.67 (0.30–1.47)
Diagnostic supportive services	0.90 (0.43–1.88)		1.14 (0.32–4.02)	0.66 (0.28–1.57)
Other	1.31 (0.68–2.54)		1.39 (0.48–4.04)	1.01 (0.47–2.15)
β per department type		–0.03 (–0.32 to 0.25)		
Work–related factors				
Weekly patient volume				
< 10 patients	1.00		1.00	1.00
10–30 patients	1.25 (0.79–1.98)		1.04 (0.49–2.23)	1.07 (0.63–1.84)
30–50 patients	1.18 (0.68–2.04)		2.05 (0.77–5.46)	1.00 (0.53–1.88)
> 50 patients	0.79 (0.50–1.26)		0.84 (0.39–1.81)	0.67 (0.38–1.18)
β per volume category		0.29 (–0.15 to 0.73)		
Night shift rotation				
No night shifts	1.00		1.00	1.00
Required to work night shifts	1.61 (1.08–2.41)	1.34 (0.10 to 2.58)	0.92 (0.49–1.71)	1.67 (1.06–2.62)
Income satisfaction				
Dissatisfied	1.00		1.00	1.00
Neutral	0.63 (0.41–0.98)		0.52 (0.27–1.03)	0.59 (0.36–0.97)
Satisfied	0.24 (0.16–0.38)		0.17 (0.08–0.35)	0.24 (0.14–0.39)
β per satisfaction level increase		–1.05 (–1.68 to –0.42)		
Research pressure				
No/Low pressure	1.00		1.00	1.00
High pressure	2.17 (1.42–3.31)	2.89 (1.71 to 4.07)	2.44 (1.24–4.79)	2.07 (1.32–3.24)
Teaching pressure				
No/Low pressure	1.00		1.00	1.00
High pressure	2.29 (1.40–5.03)	5.23 (3.45 to 7.02)	1.34 (0.42–4.31)	2.95 (1.23–7.09)

Risk factor	PFI (Primary Analysis)	SA1: PFI Continuous	SA2: Excluding Fujian Province (n = 342)	SA3: Excluding 'Other' Title (n = 814)
	Burnout OR (95% CI)	Score β (95% CI)	Burnout OR (95% CI)	Burnout OR (95% CI)
Technology factor				
AI tool use				
No	1.00		1.00	1.00
Yes	0.78 (0.57–1.07)	–0.32 (–1.26 to 0.63)	0.83 (0.50–1.38)	0.80 (0.57–1.13)

Abbreviations: OR, adjusted odds ratio; AI, artificial intelligence; CI, confidence interval; PFI, Professional Fulfillment Index; SD, standard deviation.
SA1 treat burnout and depression as continuous outcomes (linear regression); β represents change per unit increase in income satisfaction (ordinal, 1–3 scale). SA2 excludes physicians from Fujian Province (n = 619 excluded; n = 342 retained). SA3 excludes physicians with 'other' professional title (n = 147 excluded; n = 814 retained).

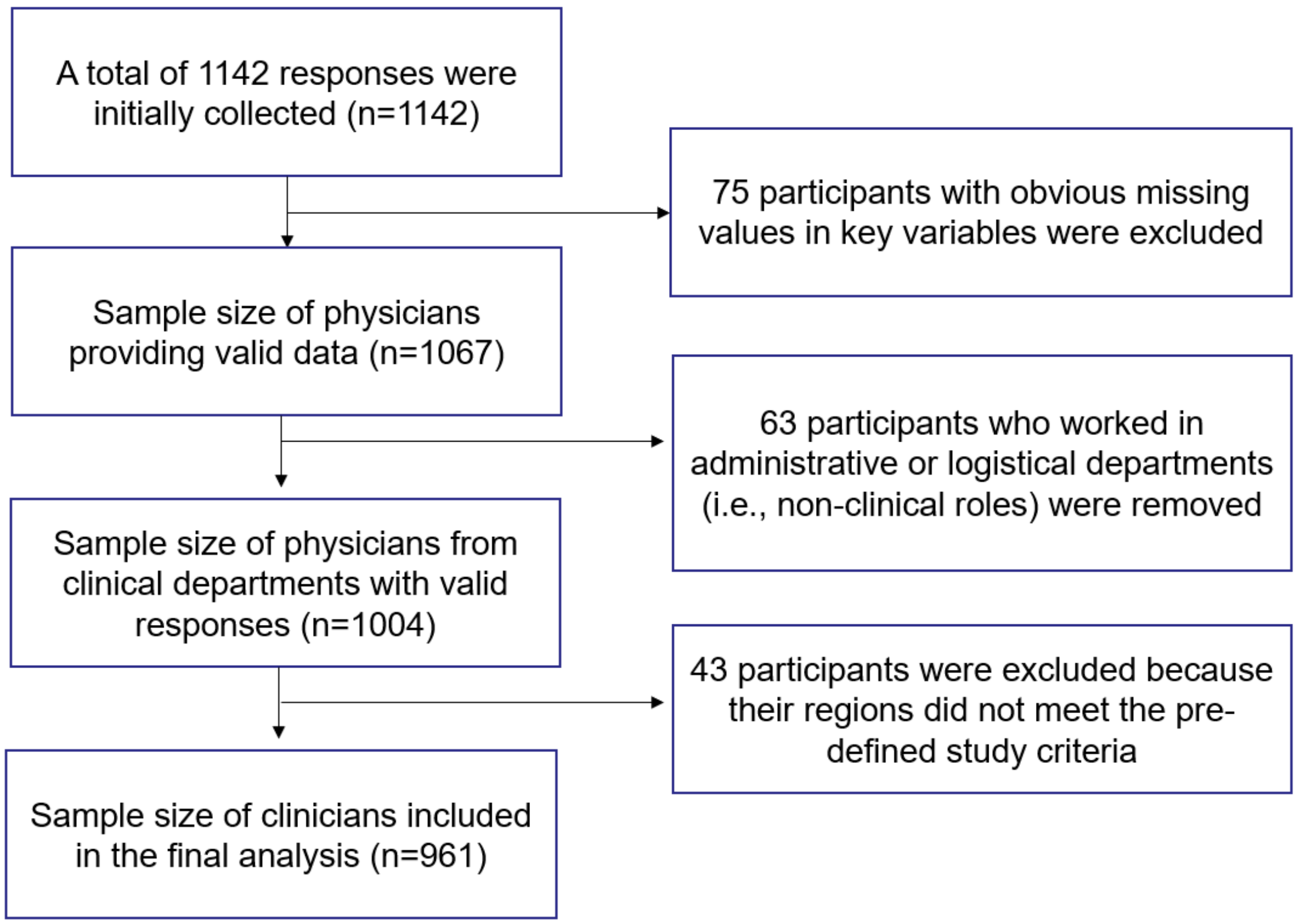

Figure S1. Flow chart
